# Comparative study of cemented versus screwed prosthesis on the marginal bone stability around dental implants

**DOI:** 10.6026/973206300220488

**Published:** 2026-01-31

**Authors:** Vijayalakshmi C.R, Animesh Barodiya, Ajay Gaikwad, Shanthana Lakshmi C.B, Santosh Kumar, Manan Shah

**Affiliations:** 1 Department of Prosthodontics, BGS Global Institute of Dental Sciences and Hospital, Bengaluru, India; 2 Department of Dentistry, Gandhi Medical College, Bhopal, India; 3 Department of Prosthodontics, School of Dental Sciences, Krishna Vishwa Vidyapeeth (Deemed To Be University), Taluka-Karad, Satara, Maharashtra, India; 4 Department of Prosthodontics Crown and Bridge and Implantology, Sri Rajiv Gandhi College of Dental Sciences & Hospital, Bengaluru, India; 5 Department of Periodontology & Implantology, Karnavati University, Uvarsad, Gandhinagar, Ahmedabad, Gujarat, India; 6 Department of Prosthodontics, Crown & Bridge, Faculty of Dental Science, Dharmsinh Desai University, Nadiad, Gujarat, India

**Keywords:** Dental implants, cemented prosthesis, screw-retained prosthesis, marginal bone loss, peri-implantitis, excess cement, biological complications

## Abstract

Cemented versus screw-retained implant crowns effects on peri-implant bone stability remain controversial despite widespread clinical use. Therefore, it is of interest to compare 38 cemented and 38 screw-retained single-implant crowns in posterior sites across 68 patients over 24 months. Screw-retained crowns showed significantly lower mean marginal bone loss (0.68 ± 0.29 mm vs. 1.12 ± 0.34 mm, p<0.001) at 24 months. Excess cement occurred in 34% of cemented cases, strongly correlating with bone loss (r=0.71, p<0.001). Screw retention demonstrates superior bone preservation and reduced biological complications, advancing evidence-based single-implant crown selection.

## Background:

Choosing the prosthetic retention mechanism is one of the most essential choices in the implant-supported restoration that combines biomechanical, biological and practical factors. Each of the two classes of cemented and screw-retained prostheses has its own benefits: Cemented restorations have better aesthetics, passive fit and less component complexity and screw-retained have guaranteed retrievability, absence of cement-related complications and controlled application of torque [1]. Although they have been used clinically over decades, there still remains controversy as to their respective effect on marginal bone stability at the implant site, which is one of the primary predictors of long-term implant success [2]. Peri- implantitis Multimodal etiologies associated with marginal bone loss around dental implants are surgical trauma, inflammatory processes at a micro-gap level, occlusal overload and peri-implantitis caused by plaque [3]. The prosthetic interface has the potential to affect these processes based on the patterns of bacterial colonization, stress distribution and residual subgingival cement [4]. The conflicting results have been reported in retrospective studies with some studies showing no significant difference on bone loss between the types of retention but others have proved excess cement to be one of the ultimate predisposing factors of peri-implant disease [5]. Systematic review indicated that screw-retained prostheses have slightly less bone loss, but study design and follow-up times did not allow definitive proposals [6]. Clinical investigations recently have shown the biological impact on residual cement; some in vitro experiments have shown cytotoxic effects of resin based luting agents on fibroblasts and osteoblasts [7]. Moreover, the subgingival microgap, which is inherent in screwed prostheses, has been depicted to retain bacterial biofilms which could stimulate inflammatory bone resorption [8]. Mechanical characteristics of these systems are also notably different, the screw-retained design creates stress concentrations at the abutment-implant interface and cemented restorations spread stress across the cement layer and the cervical area [9].

Computational models indicate that these specific load transfer processes can have different effects on crestal bone remodeling, but these effects are yet to be clinically confirmed [10]. There are a number of methodology limitations that are rife in the literature. The majority of the comparative studies are retrospective, which brings about selection bias and inconsistency in the measurement protocols [11]. The future trials are likely to involve both single and multiple-unit restorations, which will confuse the single effect of retention mechanism on bone stability [12]. Also, little studies have standardized implant position, abutment height or cementation regimens to allow simple comparisons [13]. A systematic measure of excess cement detecting procedures against later bone loss has not been done in a controlled randomized study [14]. This gap in research hinders the use of evidence-based prosthetic selection, especially with the patient group with a high risk of peri- implant disease. The clinical implications of the finding of the differential effect of retention mechanisms on the stability of marginal bone are important. As the prevalence of implants increases, peri-implantitis turns into a growing therapeutic load and impacts 20-40 percent of patients and 10-50 percent of implants [15]. In the event that cemented prostheses are proved to cause biological complication by the way of retained subgingival material, clinical policies should encompass a careful method of cement removal or prefer screw retention in high risk situations [16]. On the other hand, when there is no significant distinction, one can use aesthetic and technical factors in making decisions [17]. Therefore, it is of interest to determine the marginal bone stability, the biological complications as well as the technical outcome that could be achieved with cemented and screw-retained single-implant prostheses over a period of 24 months with the control of surgical, prosthetic and patient factors.

## Materials and Methods:

## Study design and setting:

This was a prospective, parallel-group, randomized controlled trial carried out at the Department of Prosthodontics, between March and September of 2021.

## Sample size calculation:

The analysis of power was conducted according to the expected difference in marginal bone loss (primary outcome). With a standard deviation of 0.4 mm, a clinically significant difference between the 2 inter-groups of 0.3 mm, alpha of 0.05 and power of 0.85, the study needed at least 35 prostheses in each group. The target sample size was determined to be 76 prostheses taking into consideration 10% attrition.

## Selection criteria of the participants:

Sixty-eight partially edentulous patients that needed single-implant restoration in the posterior maxilla or mandible were registered. The inclusion criteria included: Age between 30 and 75 years, healed extraction sites (more than 4 months), bone width 6 mm or higher and bone height 10 mm or higher, bone density should be D2-D3 (Lekholm and Zarb) and non-smoker or less than 5 cigarettes per day. The exclusion criteria were active periodontitis, uncontrolled type 2 diabetes (HbA1c over 7%), night guard refusal (bruxism), implant failure, radiation to the jaws and allergy to titanium or crown materials.

## Randomization and allocation:

Participants meeting the criteria were assigned to cemented and screw-retained groups randomly through computer-generated block randomization (block size of 4) stratified by the position of the jaw (maxilla/mandible) and bone density (D2/D3). The concealment of allocation was done by using sequentially numbered and sealed opaque envelopes opened once the implant was placed after confirmation.

## Implant system and surgery procedure:

White all patients were given implants of titanium (4.3 mm diameter, 10 mm length; Tapered Screw-Vent, Zimmer Biomet) with a standardized surgical template, sandblasted, acid-etched and fitted with a standard surgical template. After local anesthesia, full thickness flaps were lifted up and osteotomies were prepared using sequential drills at 800 rpm with sterile saline irrigation. The insertion torque was measured with a surgical motor that could be controlled in terms of torque (20-45 Ncm). Abutments (3 mm high) were applied and flaps re-tuned with discontinuous sutures. Conventional loading of the all implants was conducted after 12 weeks (mandible) or 16 weeks (maxilla).

## Prosthetic procedures:

All cases were chosen with standardized titanium abutments (5 mm high). In case of cemented group, they were provisional crowned to create emergence profile. Strong crowns were luted with resin-modified glass ionomer cement (RelyX Luting Plus, 3M) that was sparingly put on the intaglio surface. Extra cement was excised with the use of curettes and dental floss under magnification (4.5x surgical loupes) as soon as the cement was seated. To ensure total removal, post-cementation radiographs were done. In the case of a screw- retained group, computer-aided design/computer-aided manufacturing (CAD/CAM) zirconia crowns with inbuilt screw channels were designed. Calibration- The torque driver was adjusted to 25 Ncm and used to secure crowns. Polyethene tape and composite resin were used to close screw access holes.

## Clinical assessment:

No acute or chronic pulmonary disease is present. No lung cancer, pleural or peritoneal effusion, or nodules. Diaphragm is normal; no noise during diaphragm assessment. Nares, pharynx and throat are free of lesions.

## Diagnostic tests:

No fever or infection. No additional signs or symptoms suggestive of pneumonia. Marginal bone levels were measured on standardized periapical radiographs by means of a customized bite registration paralleling technique. Radiographs were taken at baseline (delivery of the prosthesis), 6, 12 and 24 months. The ImageJ software (v1.53, NIH) was used to analyze digital images with a calibration on the known implant diameter. The mesial and distal bone levels were determined between implant abutment junctions to the most coronal contact of bone-implant contact and averaged. The clinical parameters monitored at every visit were: probing depth (six sites per implant), bleeding on probing (BOP), plaque index and presence of suppuration. Peri-implant mucositis was considered to be BOP with bone loss less than 2 mm; peri-implantitis BOP with bone loss more than 2 mm and progressive bone loss.

## Excess cement detection:

Probing using a plastic instrument was conducted to identify the residual of subgingival cement at 6-month follow-up. Hard and rough deposits were made beneath the mucosal margin, which were identified as positive. Sites were then covered with chlorhexidine gel applied locally and ultrasonic debridement.

## Technical complications:

The complications of the prosthetics were observed during the study period that includes: Screw loosening, crown de-cementation, chipping of porcelain and fracture of the abutment. Retrievability was measured in terms of being able to take the prosthesis off without any kind of damage to undertake any biological or technical procedure.

## Statistical analysis:

The analysis of data was made with SPSS 27.0. Mean, standard deviation was used as a way of expressing continuous variables. Parametric data were compared using independent samples t-test to compare the inter-group differences. Fisher and Chi-square tests were used to determine categorical variables. ANOVA measured bone loss after time. Pearson correlation was used to test the correlation between excess cement and bone loss. Kaplan-Meier analysis was not done because the survival was 100 percent. The p-value was set to be 0.05, which was the level of statistical significance.

## Results:

Scientific evidence shows that 76 implants were applied to 68 patients (36 female, 32 male; mean age 54.8 ± 9.3 years). A patient lost to follow-up due to moving to a different location was lost in the cemented group, so final analysis would be done on 37 prostheses. There were no significant group differences in terms of age, sex distribution, jaw location, bone density or insertion torque (Table 1). Mean abutment height was equalized to 5 mm in all the cases. The difference in means of marginal bone loss in each group at each time point was significant (Table 2). Cemented prostheses at the age of 6 months showed bone loss of 0.48 + 0.18 mm compared to screw-retained prostheses of 0.31 + 0.14 mm (p<0.001). This difference grew with time: At 12 months, the value was 0.82 0.26 mm and 0.51 0.22 mm (p<0.001) and at 24 months, 1.12 0.34 mm and 0.68 0.29 mm (p<0.001). ANOVA of repeated measures affirmed the existence of significant group-by-time interaction (F=34.7, p<0.001), which showed divergent bone loss curves. The rate of bone loss in the screw-retained group (0.03 mm/month) was linear compared to cemented prostheses (0.06 mm/month) whose bone loss increased rapidly with time (between 6-12 months) and then levelled off. The bone loss in cemented prostheses was found to be 0.19 mm in bone D3 than in bone D2 (p=0.012), indicating that bone density alters the effect of retention mechanism. At 6 months examination, subgingival cement remnants were found in 13 of 38 cemented prostheses (34.2). The sites that had excess cement showed much bone loss of 24 months (1.34 + 0.31 mm) when compared to cement-free sites (0.98 + 0.29 mm, p=0.003). Pearson correlation showed that there was a strong positive correlation between bone loss and the presence of cement (r=0.71, p<0.001). The cemented group had more biological complications than the non-cemented group (Table 3). The occurrence of peri-implant mucositis was 8 cemented (21.1) compared to 3 screw-retained (7.9, p=0.089). The peri-implantitis was diagnosed in 1 cemented case (2.6) with progressive bone loss and suppuration and necessitating surgical intervention. No screw-retained prostheses came up with peri-implantitis. At 24 months, bleeding on probing was significantly more common in cemented group (28.9% versus 13.2% of sites p=0.041). The mean probing depths were similar (at baseline) but separated over time, where cemented prostheses indicated 3.4 ± 0.7 mm compared to 2.9 + 0.5 mm at 24 months (p=0.002). The study showed that all the prostheses were still in use after the study period and that 100 percent of the subjects in both groups survived. The qualitative differences between technical complications are; three cemented crowns needed re-cementation because of the presence of de-cementation at 14-18 months and two screw- retained prostheses had their screws loosened and tightened to 25 Ncm at routine maintenance. There was one porcelain chipping per group in both composite resin repaired prostheses. In two instances of mucositis that needed flap debridement, retrievability was beneficial because non-destructive access was preferable to enable biological treatment in the screw-retained group. The cemented group had 0.28 mm more bone loss in posterior maxillary location than mandibular locations (p=0.033), which may be explained by the more difficult removal of cement in less reachable places. There was no such difference in the screw-retained group (p=0.523). Both types of cemented prostheses resulted in bone loss which was faster in the smokers (n=6) but not significantly different between groups (1.42 0.38 mm vs. 0.81 0.31 mm in non-smokers, p=0.014).

## Discussion:

The presented prospective randomized controlled trial offers strong support that the use of prosthetic retention mechanism strongly affects the peri-implant marginal bone stability during 24 months. The overall result shows that screw-retained prostheses retain marginal bone better than cemented restorations and mean difference is 0.44 mm after 24 months. Although this absolute difference can be considered small, it is a 39% decrease in bone loss that can have dramatic implications on long-term implant prognosis, especially in patients with limited bone volume or high biological risk [18]. The pattern of bone loss in the cemented prostheses that was observed had the accelerated bone resorption rate in the first 12 months, which is strongly associated with the existence of subgingival cement residues. The 34.2% excess cement rate was detected in our study in agreement with the past findings that reported residual cement as a common complication [19]. Interestingly, sites that had cement depiction had 37 percent higher bone loss as compared to the sites that had no cement, which highlights the bio insult of retained foreign material. Histological studies have also revealed that resin-based cements produce inflammatory effects and osteoclastic activity which may be the cause of the local bone resorption pattern evident [20]. The correlation coefficient (r=0.71) between the presence of cement and the magnitude of bone loss reveals strong clinical evidence of causal relationship between the two variables and the hypothesis that cement elimination is a critical determinant of peri-implant health is confirmed. No evidence of microgap leakage of the screw-retained prostheses, which has been previously implicated in bacteria leakage and inflammatory bone loss, could be found as a drawback in our cohort [21]. The low rate of bone loss (0.68 mm) is in good comparison with the historical controls and indicates that the current conical connections and appropriate torque application successfully seal the interface [22]. The higher bleeding on probing and deeper probing depths in the cemented prosthetic are probably due to the combined effects of remaining cement and inflammatory infiltrate, which is in line with the microbiological data that show a higher pathogen load in cement-associated lesions [19]. On the contrary, the screw-retained group did not show any changes in the soft tissue parameters, which indicates that retrievability allows them to maintain and intervene early before the disease progresses. Technical issues were more represented by the variations between the groups and they are part of the system nature. The three incidences of cemented prosthesis failure were witnessed in cemented prostheses even after the use of resin-modified glass ionomer reported to have long-term retentive force implying that the occlusal force and preparation geometry could affect long-term stability [23]. The two cases of loosening screws in the screw-retained group were reported at the time of routine maintenance and could easily be handled by retightening, which depicts the feasible benefit of retrievability. This non-destructive accessibility of the prosthesis that facilitates non-destructive biological treatment of two mucositis cases also demonstrates the advantage as compared to cemented prostheses where intervention would involve cutting of crowns or sacrifices. Bone density has become an adjusting factor and D3 bone demonstrates increased bone loss in cemented prostheses. This communication probably signals the impairment of vascularity and decreased remodeling potential of low-density bone making it more susceptible to inflammation caused by cement [24].

The lack of this effect in screw-retained prosthesis predicts that design-specific stress distribution might not be as critical as prevention of biological insults in compromised locations. Likewise, the posterior maxilla also exhibited a higher loss of bone with cemented prostheses, which may likely be explained by the fact that this part of the anatomy is not easily visible and accessed by hand to completely remove the cement [25]. This investigation has its strengths in the form of randomized prospective design, standardized protocols and the incorporation of both radiographic and clinical parameters. Our allocation method also equalised confounding factors such as bone density and jaw location unlike the retrospective analyses, which are prone to selection bias [26]. Single implant system was used, which removed surface topography as a variable, so that only the effect of retention mechanism is isolated. Digital subtraction analysis and radiographic standardization improved the accuracy of the measurement compared to that performed by visual estimation used in most past studies [21]. A number of limitations need to be mentioned. Although the 24-month follow-up is adequate in terms of assessing the initial remodeling of bones, it does not assess the long-term stability after the initial healing and adaptation processes. A longer lag period (5-10 years) would help to understand the continuation of divergence or stabilization [26]. The single operator protocol is consistent and can restrict the generalizability to different levels of clinical skill, specifically in cement removal technique, which has a vital impact on the outcome. We used the tactile probing as the detection technique to determine excess cement instead of the microscopic or surgical confirmation, which may have underestimated the remaining material in the deep areas of the subgingival sites [27]. The standardized height of abutment (5 mm) might not respond to clinical cases that involve deeper placement due to aesthetic factors where the cement removal will become exponentially difficult. Ways of future research are to study the results of different abutment height and subgingival depth. Also, our cohort restricted itself to patients that have severe bruxism and uncontrolled systemic disease and thus restricted itself to medically compromised groups. The size of the sample, though sufficient in the primary outcome analysis, had a short power to detect uncommon complications such as peri- implantitis. Clinically, these results support the re-evaluation of the algorithms of prosthetic choice. The old paradigm of cemented restorations to be selected in esthetic areas due to the advantageous appearance of the emergence profile has to be balanced with the biological risks, especially in case of need of subgingival margins [28]. Screw-retained designs with designed angulated screw channels now provide similar aestheticism but eliminate cement-effect issues, which may tip the balance to the advantage side [29]. Screw retention would be obviously beneficial in the case of posterior areas where retrievability and access to maintenance are of primary importance. Economic factors are worth being discussed as well. Although cemented prostheses have lower laboratory costs, the high prevalence of biological complications that require management could balance the initial savings [30]. Treatment cost of peri-implantitis, especially surgical, is significantly high compared to the incremental cost of screw-retained components. Complication rates and treatment costs would be used to create a formal cost-effectiveness analysis that would guide the use of healthcare resources. Patient-reported outcomes are not formally measured in this trial, but this is a significant dimension. Anecdotally, patients indicate that screw access holes are aesthetic breakdowns, but this issue can be addressed by appropriate positioning and opaque sealing material [31]. Probably the inconvenience of the events of decementation that need to visit the emergency, rather than the existence of a minute access channel has a greater effect on satisfaction. Validated quality-of-life measures should be incorporated in future studies to ensure that these patient-centered views are holistic. Finally, this randomized trial shows that screw-retained prostheses are better in preserving marginal bone and have less biological complications compared to cemented restorations in 24 months. Excess cement in a third of cemented cases is strongly correlated with loss of bone and provides a definite biological rationale of the mechanism of retention when choosing between the two. Although the two designs provide equal survival rates, the technical strengths of retrievability and improved peri-implant health justify the use of screw retention over the alternative in areas where bone preservation and maintenance is the main concern. The results must be incorporated into clinical practice and promote careful cement removal measures, in cases when cemented prostheses are inevitable.

## Conclusion:

Screw-retained prostheses showed 39 percent less bone loss and significantly reduce biological complication rates than cemented restorations at 24 months. Residual cement is an etiological factor as it is detected by a high correlation with the excess cement in 34% of cemented cases and high bone loss. Although the success of both retention systems was 100 percent with respect to prosthetic survival, the ability to retrieve and predictable maintenance of screw-retained designs have different clinical benefits.

## Figures and Tables

**Table 1:** Baseline demographics and surgical parameters

**Parameter**	**Cemented (n=38)**	**Screw-Retained (n=38)**	**p-value**
Age (years)	55.2 ± 9.8	54.4 ± 8.9	0.712
Sex (F/M)	20/18	18/20	0.639
Maxilla/Mandible	21/17	19/19	0.639
Bone density D2/D3	22/16	24/14	0.523
Insertion torque (Ncm)	34.8 ± 7.2	35.6 ± 6.8	0.623
Abutment height (mm)	5.0 ± 0.2	5.0 ± 0.2	0.852
No statistically significant
differences were observed between
groups at baseline

**Table 2:** Marginal bone loss at different time intervals

**Time Point**	**Cemented (mm)**	**Screw-Retained (mm)**	**Mean Difference (95% CI)**	**p-value**
6 months	0.48 ± 0.18	0.31 ± 0.14	0.17 (0.09-0.25)	<0.001*
12 months	0.82 ± 0.26	0.51 ± 0.22	0.31 (0.20-0.42)	<0.001*
24 months	1.12 ± 0.34	0.68 ± 0.29	0.44 (0.30-0.58)	<0.001*
*Statistically significant difference
(p<0.05). Bone loss was significantly
greater in the cemented group
at all-time intervals

**Table 3:** Biological and technical complications at 24 months

**Complication**	**Cemented (n=37)**	**Screw-Retained (n=38)**	**p-value**
Excess cement detection	13 (35.1%)	0 (0%)	<0.001*
Peri-implant mucositis	8 (21.6%)	3 (7.9%)	0.089
Peri-implantitis	1 (2.7%)	0 (0%)	0.311
Bleeding on probing (sites %)	28.9 ± 8.4	13.2 ± 5.1	0.041*
Mean probing depth (mm)	3.4 ± 0.7	2.9 ± 0.5	0.002*
Crown decementation	3 (8.1%)	0 (0%)	0.081
Screw loosening	0 (0%)	2 (5.3%)	0.149
Porcelain chipping	1 (2.7%)	1 (2.6%)	0.984
*Statistically significant difference
(p<0.05). Cemented prostheses showed
higher biological complication rates
